# On the Mechanism of Genipin Binding to Primary Amines in Lactose-Modified Chitosan at Neutral pH

**DOI:** 10.3390/ijms21186831

**Published:** 2020-09-17

**Authors:** Chiara Pizzolitto, Michela Cok, Fioretta Asaro, Francesca Scognamiglio, Eleonora Marsich, Francesco Lopez, Ivan Donati, Pasquale Sacco

**Affiliations:** 1 Department of Medical, Surgical, and Health Sciences, University of Trieste, piazza dell’Ospitale 1, 34127 Trieste, Italy; CHIARA.PIZZOLITTO@phd.units.it (C.P.); fscognamiglio@units.it (F.S.); emarsich@units.it (E.M.); 2 Department of Life Sciences, University of Trieste, via Licio Giorgieri 5, 34127 Trieste, Italy; mcok@units.it (M.C.); idonati@units.it (I.D.); 3 Department of Chemical and Pharmaceutical Sciences, University of Trieste, via Licio Giorgieri 1, 34127 Trieste, Italy; fasaro@units.it; 4 Department of Agricultural, Environmental and Food Sciences (DiAAA), University of Molise, Via De Sanctis, I-86100 Campobasso, Italy; lopez@unimol.it

**Keywords:** primary amines, genipin as ligand, lactose-modified chitosan, neutral pH, mechanism of reaction

## Abstract

The present manuscript deals with the elucidation of the mechanism of genipin binding by primary amines at neutral pH. UV-VIS and CD measurements both in the presence of oxygen and in oxygen-depleted conditions, combined with computational analyses, led to propose a novel mechanism for the formation of genipin derivatives. The indications collected with chiral and achiral primary amines allowed interpreting the genipin binding to a lactose-modified chitosan (CTL or Chitlac), which is soluble at all pH values. Two types of reaction and their kinetics were found in the presence of oxygen: (i) an interchain reticulation, which involves two genipin molecules and two polysaccharide chains, and (ii) a binding of one genipin molecule to the polymer chain without chain–chain reticulation. The latter evolves in additional interchain cross-links, leading to the formation of the well-known blue iridoid-derivatives.

## 1. Introduction

Lactose-modified chitosan, commercially known as CTL, is a bioactive polysaccharide derived from natural resources. While containing a chitosan backbone with β-1 → 4 linked d glucosamine units (deacetylated units, D) interspersed by residual *N*-acetyl-d-glucosamines (acetylated units, A), CTL bears lactitol moieties inserted on some D units by means of an *N*-alkylation reaction [1]. Molecular dynamics simulations run on decamers showed the propensity of the modified chitosan to assume an extended 3_2_ helical geometry at low ionic strength and a 2_1_ geometry when electrostatic interactions are screened [2]. The presence of the flexible flanking groups endows lactose-modified chitosan with peculiar physical-chemical properties [3], which range from a complete solubility at all pH values to the formation of synergistic mixtures with polyanions [4,5]. Given its intriguing biological properties [6], lactose-modified chitosan has been explored as active component in tissue engineering applications for cartilage, bone, and neural tissues [7,8,9].

Due to its polycationic nature and the presence of primary amines from the unreacted glucosamine units, lactose-modified chitosan could form physical and chemical hydrogels, exploiting well known approaches developed for its parent compound chitosan [10,11]. Lactose-modified chitosan forms, in the presence of boric acid, transient networks [12,13] showing strain-hardening and self-healing properties [14]. However, for long-lasting applications in the field of biomaterials, the introduction of chemical cross-links is beneficial to avoid the continuous rearrangement of the network. Chemical cross-linkers, such as glutaraldehyde, are often used to increment chitosan’s stiffness and strength [15]. However, concerns on cytotoxicity of the cross-linker limit its use for biomedical applications. For this reason, covalent cross-linkers of natural origin have been sought.

Genipin (methyl-1-hydroxy-7-(hydroxymethyl)-1,4a,5,7a-tetrahydrocyclopenta [c]pyran-4-carboxylate) is a colorless cyclopentapyrane-type monoterpenoid (iridoid). Genipin is produced from the hydrolysis of geniposide extracted from the fruits of *Gardenia Jasminoides Ellis*, traditionally used in oriental medicine for the treatment of inflammation, headache, and hepatic disorders. Genipin is largely used as reticulating agent because it is less toxic than other covalent cross-linkers: approximately 1000 times lower then glutaraldehyde [16]. Genipin has been used in tissue fixation to cross-link collagen and gelatin and in foodstuff to cross-link soy proteins [17]. 

One of the fundamental characteristics of the reaction between genipin and primary amines, in the presence of oxygen, is the formation of a blue color [18]. The binding of genipin by methylamine has been studied from an experimental and theoretical point of view. This led to propose different molecular structures as blue pigments and intermediates [18,19]. The binding of genipin to chitosan as polyelectrolyte bearing primary amines, and its applications for tissue engineering, has also been largely investigated [20,21]. Using UV-VIS spectroscopy, Butler et al. [17] proposed a mechanism based on two reaction pathways (Figure S1). A first and faster reaction involves one genipin and one amino group on chitosan, evolving further into interchain association due to a reaction between bound genipin residues. The slower second reaction predicts a genipin molecule bridging to chitosan chains via contemporary opening of the dihydropyran ring and the conversion of the ester bond into an amide. Despite all these efforts, the scenario of polymer reticulation by genipin still shows contrasting mechanistic hypotheses. In addition, as long as chitosan is concerned, all the aforementioned experiments were carried out in acidic pH.

The present work aims at elucidating the mechanism of genipin binding to primary amines in physiological conditions using lactose-modified chitosan as polysaccharide. Small molecules such as glucosamine and ethylamine were used for comparison. The study entails different spectroscopic techniques, namely UV-VIS and circular dichroism, and computational analyses. The combination of this information allows proposing a mechanism for genipin binding in the presence and in the absence of oxygen, which was confirmed by kinetics analyses. To the best of the authors’ knowledge, this is the first contribution in which circular dichroism is used extensively to screen the binding of genipin. 

## 2. Results

### 2.1. Auto Reaction of Genipin

**Figure 1:**
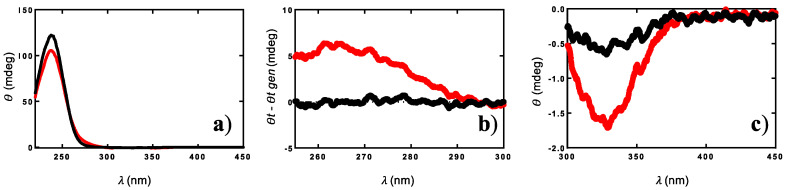
Genipin incubated at 37 °C in PBS (pH 7.4) at time 0 (black) and after 6 h (red). (**a**) Overall CD spectrum. Magnification of (**a**) in the range 250–300 nm (**b**) and 300–450 nm (**c**). In (**b**), the red curve represents the difference between the CD spectrum at time *t* and the spectrum of the sole genipin at the concentration calculated from the CD signal at 240 nm.

Genipin is known to bind molecules bearing primary amino groups, leading to the formation of blue-colored solutions. However, genipin itself, when maintained at pH 7.4 and at 37 °C, undergoes auto reactions that can be monitored by circular dichroism (Figure 1a–c). 

The incubation of genipin for 6 h accounts for a 14% decrease in its concentration in solution, as calculated from the CD signal at 240 nm (Figure S3). In the same time frame, two additional CD signals are detectable: a negative peak at 330 nm and a positive one at 265 nm. The latter emerges when the spectrum of the sole genipin at the concentration determined by the ellipticity at 240 nm is subtracted from the experimental spectrum at the same time point. Two isosbestic points can be detected, namely at around 297 and 258 nm. While genipin itself reacts, to some extent, in the presence of oxygen also in the absence of primary amino groups, no blue color develops within 24 h of incubation. 

### 2.2. Binding of Genipin to Small Molecules Containing Amino Groups

**Figure 2:**
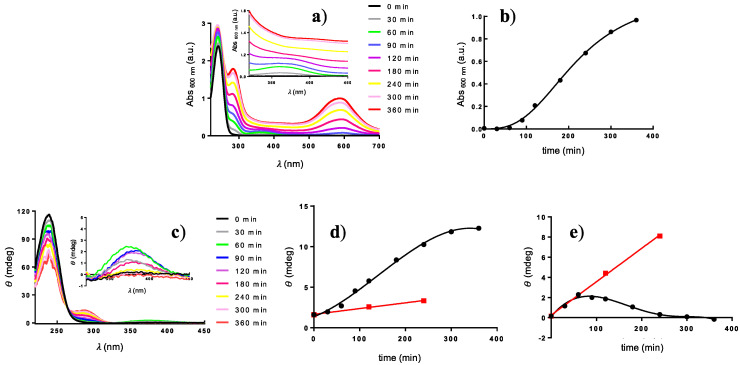
UV-VIS (**a**) and CD (**c**) spectra of glucosamine treated with genipin at 37 °C recorded at different time intervals. Inset: magnification of the spectrum in the range 320–450 nm. (**b**) Time dependence of the UV-VIS signal recorded at 600 nm. (**d**) and (**e**) Time dependence of the CD signal recorded at 280 nm (**d**) and 380 nm (**e**), respectively, in the presence of oxygen (black dots) or in oxygen-depleted conditions (red squares). Lines are drawn to guide the eye. Conditions: PBS at pH 7.4. Primary amine concentration = 1 mM. Genipin concentration = 0.37 mM.

Glucosamine is incubated with genipin at 37 °C and UV-VIS and CD spectra are recorded at different time-points in the presence of oxygen (Figure 2a–e). 

The UV spectra are difficult to interpret (Figure 2a). Upon increasing the incubation time, the signal belonging to genipin, i.e., the peak at 240 nm, increases and peaks at around 280 and 600 nm emerge. In addition, a peak at 380 nm appears for the first time-points analyzed and at a later incubation stage, it becomes indistinguishable from the tails of peaks centered at 280 and 600 nm or other signals in a similar wavelength range.

The CD spectrum of genipin/glucosamine mixture is recorded as a function of incubation time at 37 °C in PBS at pH 7.4 (Figure 2c–e). The obtained data show that the peak at 240 nm decreases over time, giving an estimate of genipin consumption due to the reaction with glucosamine (Figure 2c). The peak at 280 nm, already detected in the UV spectrum, is also evident (and positive in sign) in the CD spectrum, thus it stems from the formation of a chiral molecule in solution (Figure 2d). The peak at 600 nm, associated with the blue color typically found for genipin treated with primary amines, is not visible in the CD spectrum up to 24 h of incubation time (data not reported). Furthermore, CD spectra efficiently monitor the increase over time of the (positive) band at around 380 nm (Figure 2e).

When glucosamine and genipin are incubated in the presence of oxygen, the ellipticity of the peak at around 280 nm shows a sigmoidal increase over incubation time. At variance, the ellipticity of the peak at 380 nm displays a non-monotonic trend: a first increase of the signal for the first 90 min is in fact followed by a marked decrease afterwards. Indeed, the signal completely vanishes after 6 h of incubation. This behavior may be safely correlated with the time-increase of the UV-VIS signal at 600 nm: negligible for the first 90 min and rapidly increasing thereafter.

The interaction between glucosamine and genipin is also followed in oxygen-depleted conditions for 4 h (Figure 2d,e). The CD band at 280 nm almost vanishes when oxygen is depleted, while under the same conditions, the ellipticity of the peak at 380 nm reverts to a linear increase over the first 4 h. No blue color develops after 4 h of incubation under oxygen-depleted conditions.

**Figure 3:**
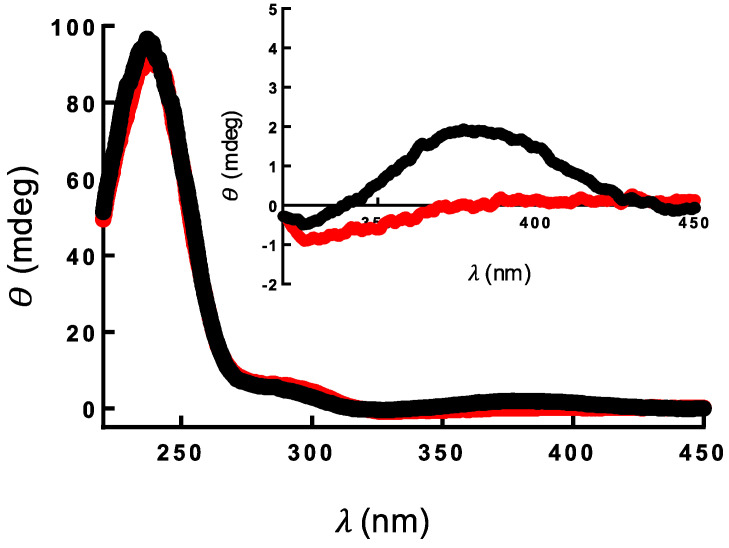
CD spectrum of genipin (0.37 mM) incubated with glucosamine (black curve) and ethylamine (red curve), respectively, for 2 h at 37 °C in PBS at pH 7.4. Inset. Magnification of the range 320–450 nm. In both cases, the concentration of amino groups was 1 mM.

Ethylamine is used as an achiral primary amine model and the binding to genipin is analyzed by CD spectroscopy in the presence of oxygen. While the ellipticity of the band at around 280 nm increases over time, the signal at around 380 nm cannot be detected (Figure 3). 

Since it is well known that genipin treated with ethylamine displays a UV peak at around 380 nm [19], the CD band stems from a molecule that either shows no chiral centers in the genipin derived moiety or it is present in a racemic mixture. 

### 2.3. Binding of Genipin to CTL at Neutral pH

**Figure 4:**
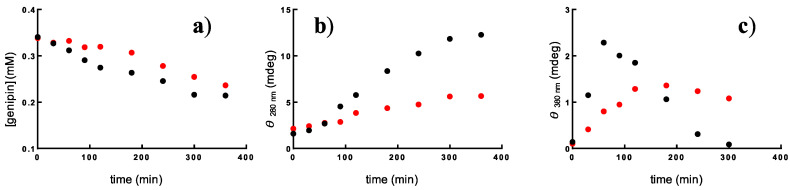
(**a**) Decrease of genipin concentration as measured from the ellipticity at 240 nm (Figure S3) and increase of the ellipticity at 280 nm (**b**) and at 380 nm (**c**) for glucosamine (black) and CTL (red). In both cases, the concentration of primary amino groups was 1 mM and the incubation was performed in the presence of oxygen at 37 °C in PBS with pH 7.4.

We now consider lactose-modified chitosan (CTL) as a polysaccharide containing primary amino groups to investigate genipin binding. CD spectra are measured for genipin incubated with CTL at a concentration equaling the amount of primary amino groups used in the case of glucosamine. The CTL-genipin system shows a time-dependent decrease in the ellipticity at 240 nm and an increase in the ellipticity for both peaks at around 280 and 380 nm, respectively. However, marked deviations can be observed compared with the case of glucosamine/genipin (Figure 4). 

The decrease of genipin concentration in solution with CTL is slightly slower than the one with glucosamine, although the concentration of free primary amino groups is the same. This behavior can be ascribed to the overall increase in viscosity due to CTL reticulation, thus likely lowering genipin accessibility to the primary amino groups. The same physical-chemical considerations account for the lower initial rate of development of the signal at 280 and at 380 nm, respectively, with respect to glucosamine. The latter signal displays a slight non-monotonic trend over time and the lower peak at 600 nm (Figure S6), with respect to glucosamine, correlates with an impairment of a molecular chain–chain association. This can be seen also in the Supplementary Materials section (Figure S4) where the time dependence of the ellipticity at 380 nm is reported for three different concentrations of CTL. The increase in polymer concentration—and the consequent increase in viscosity—causes the impairment of the conversion of the peak at 380 nm into the UV-VIS signal at 600 nm. Indeed, for CTL concentrations of 3.75 and 7.5 g/L, respectively, there is no decrease of the peak at 380 nm after 6 h of incubation with genipin (Figure S4a). At the same time, the UV-VIS signal at 600 nm is basically independent between the two concentrations (Figure S4b in Supplementary Materials).

The incubation of CTL/genipin mixture in oxygen-depleted conditions has no-effect on the initial rate of development of the peak at 380 nm (Figure S5). However, after four hours, the CD signal at 380 nm is higher than the one detected for the incubation in the presence of oxygen. When the incubation in the oxygen-depleted condition is extended up to 24 h, the blue color of the solution is much less marked than the one measured in the presence of oxygen (Figure S4c). Finally, once the sample is exposed to oxygen after 24 h of incubation in oxygen-depleted conditions, the UV signal at 600 nm is not recovered (not reported).

### 2.4. Effect of Genipin and CTL Concentration on Binding Kinetics

**Figure 5:**
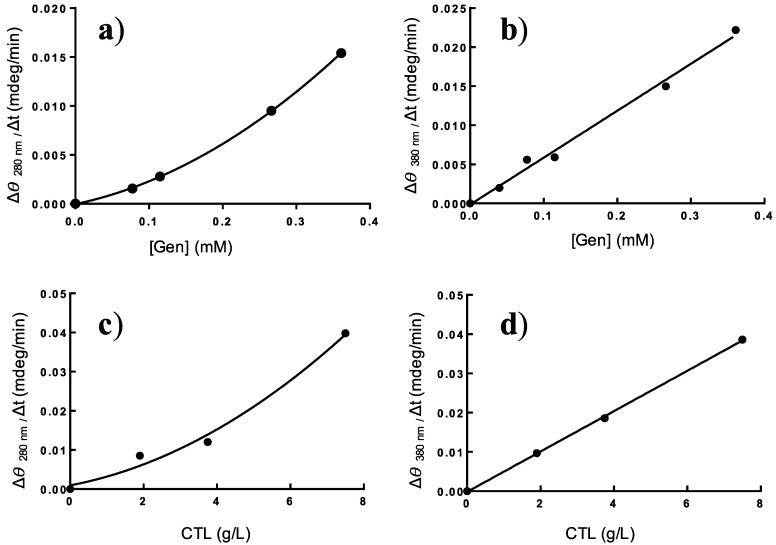
(**a**,**b**) Dependence of the initial rate of development of peaks at 280 nm (**a**) and 380 nm (**b**), respectively, from genipin concentration. CTL concentration was equal to 3.75 g/L. (**c**,**d**) Dependence of the initial rate of development of peaks at 280 nm (**c**) and 380 nm (**d**), respectively, from CTL concentration. Genipin concentration was equal to 0.37 mM. In (**a**) and (**c**), the black line represents the best-fit of the experimental data according to a parabolic equation $y=ax^{2}+bx$ (*R*^2^ > 0.985). In (**b**) and (**d**), the black line represents the best-fit of the experimental data according to a linear equation y=ax (*R*^2^ > 0.99). In both cases, the samples were incubated in the presence of oxygen at 37 °C in PBS at pH 7.4.

The effect of CTL and genipin concentration on the initial rate of development, ν, referred to the CD signals at 280 and 380 nm, respectively, provides information on the mechanism and kinetics of the binding among the two. The initial rate ν was evaluated as (Equations (1) and (2)) (Figure 5):

(1) $$v_{280\mathrm{nm}}=\frac{d\left[280\;\mathrm{nm}\right]}{dt}\propto \frac{d\left(\theta_{280\mathrm{nm}}\right)}{dt}$$

(2) $$v_{380\mathrm{nm}}=\frac{d\left[380\;\mathrm{nm}\right]}{dt}\propto \frac{d\left(\theta_{380\mathrm{nm}}\right)}{dt}$$

where [280 nm] and [380 nm] represent the molar concentration of the species providing the CD signal at 280 and 380 nm, respectively, while *θ*_280nm_ and *θ*_380nm_ represent the ellipticity at 280 and 380 nm, respectively. 

The peak at 280 nm shows a kinetic of the second order both in genipin and *CTL* concentration (Equation (3)):

(3) $$\nu_{280\mathrm{nm}}\alpha {\left[Genipin\right]}^{2}{\left[CTL\right]}^{2}$$

At variance, the peak at 380 nm displays a kinetic of the first order both in genipin and *CTL* concentration (Equation (4)):

(4) $$\nu_{380\mathrm{nm}}\alpha \left[Genipin\right]\left[CTL\right]$$

## 3. Discussion

The binding of genipin to primary amino groups has been largely exploited for the preparation of functional structures in drug delivery and biomaterials development [22,23,24,25,26,27,28]. Chitosan is a polysaccharide bearing primary amino groups that has sprouted notable interest for the preparation of hydrogels and scaffolds in which genipin replaces more cytotoxic molecules such as glutaraldehyde [16,29]. Although the mechanism of genipin binding by chitosan has already been reported, it is strictly valid in acidic conditions [17,30]. Moreover, as chitosan is usually solubilized in acidic media, the amount of genipin needed for the reticulation can be as high as 500 mM [31].

CTL replaces some of the primary amino groups of chitosan with secondary ones originated from flanking flexible side chains inserted onto the chitosan backbone [3]. It follows that CTL is soluble at all pH values [1], and it has the advantage of bearing residual primary amino groups which could be exploited for chemical reticulation with genipin. Given the pKa values of CTL [3], most of the primary amino groups are deprotonated at pH 7.4 [11], and genipin binding occurs at concentrations of reticulating agent as low as 0.13 mM.

Prior to the binding by CTL, the behavior of genipin alone and in the presence of small molecules bearing primary amino groups is considered. 

**Figure 6:**
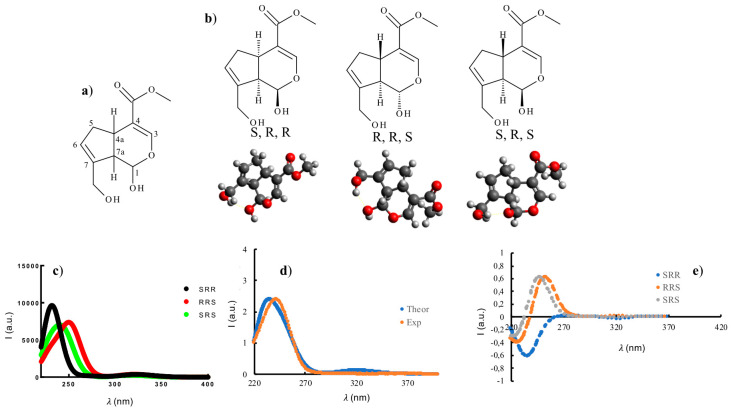
(**a**) Schematic representation of genipin. (**b**) Stereoisomers of genipin with the optimized modeling of the structure. The geometrical optimization of the molecule was performed using Avogadro software applying MMFF94 force field method. (**c**) Computed UV-VIS spectra for the stereoisomers reported in (**b**). (**d**) Comparison between the calculated UV-VIS spectrum, obtained from the combination of the three spectra in (**c**) and the experimental one. (**e**) Calculated CD spectra for the stereoisomers in (**b**). UV-VIS and CD spectra were calculated with the ORCA package.

Genipin shows a UV peak and a positive CD band at around 240 nm (Figure 1 and Figure 6), strongly dependent on its concentration in solution (Figure S3). Hence, not all the 8 possible stereoisomers of genipin are present in solution. In line with Di Tommaso et al. [32], here we assume that the main stereoisomer present is the (S,R,R) one. However, the latter would give rise to a negative CD peak as computed in Figure 6. 

We therefore assume the presence of two other stereoisomers, with the same configuration at C7a and the inverted configuration at C4a, i.e., (R,R,S) and (S,R,S). We further assume that the sum of the latter two equals the amount of the (S,R,R) stereoisomer. The computed UV-VIS spectra for the three stereoisomers, i.e., (S,R,R), (R,R,S,) and (S,R,S), are reported in Figure 6 and their combination compares rather well with the experimental data for genipin. The computed CD spectrum of the stereoisomers (R,R,S) and (S,R,S) shows a positive band at around 240 nm. Therefore, the combination of the three stereoisomers is expected to bring about a positive CD signal as found experimentally (Figure 1). 

**Scheme 1:**
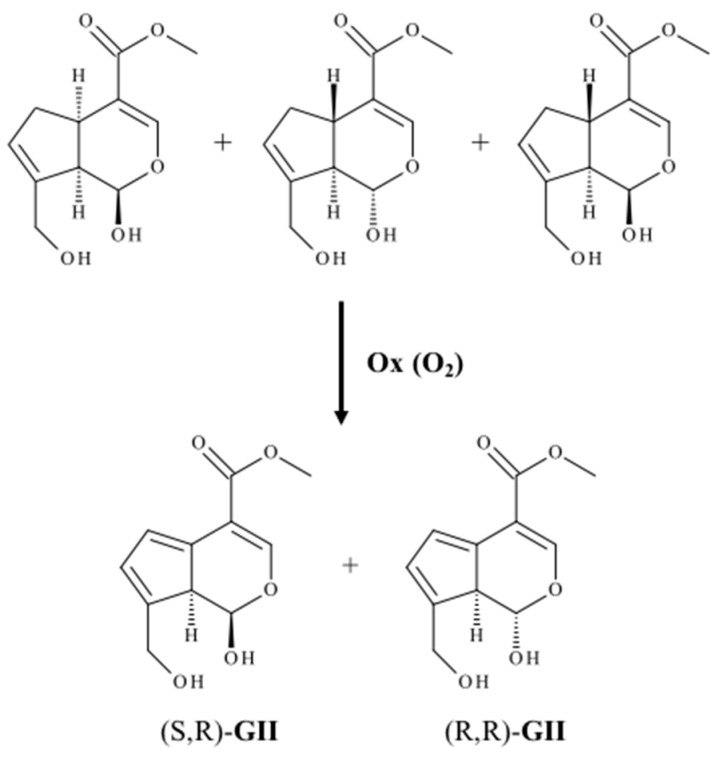
Proposed reaction scheme for genipin alone incubated at 37 °C in PBS at pH 7.4.

When genipin is incubated in PBS (pH 7.4) at 37 °C in the presence of oxygen, two bands emerge in the CD spectrum, at 265 nm (positive) and 330 nm (negative), respectively (Figure 1). However, no blue color develops over 24 h of incubation. We propose that the presence of oxygen leads to an oxidation of genipin into compound **GII**, which shows the presence of two chiral centers accounting for the CD signal (Scheme 1). 

Based on computational evaluations, both stereoisomers, i.e., (S,R)-**GII** and (R,R)-**GII**, show a UV-VIS spectrum with two peaks in the range of 260–280 nm and 320–340 nm; the first one positive and the latter one negative at the CD spectrum (Figure S7). 

**Scheme 2:**
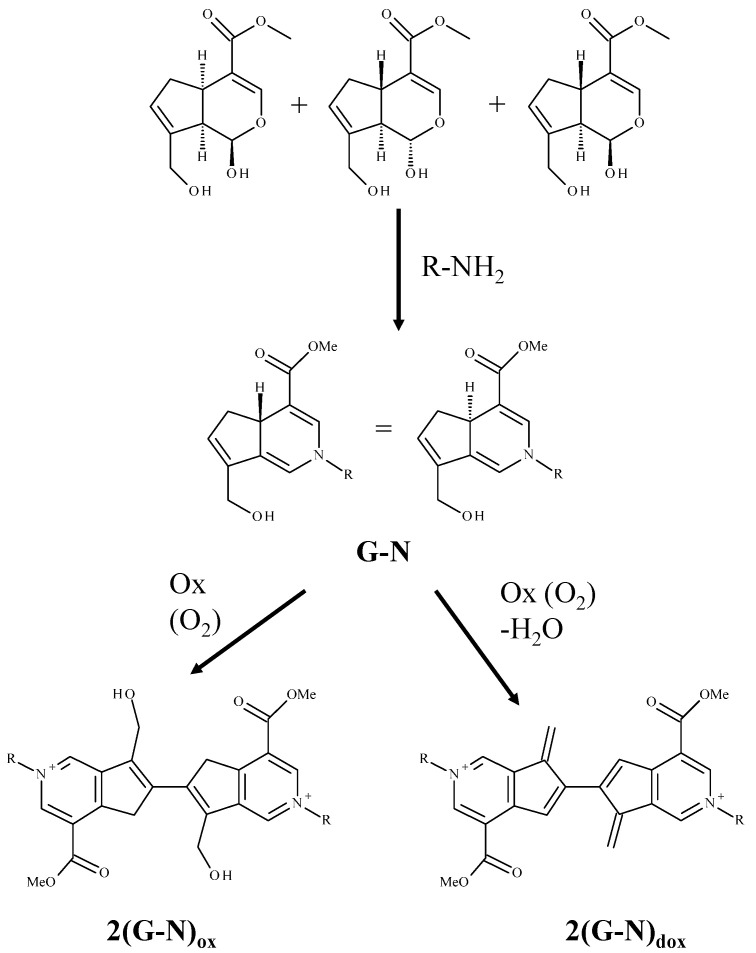
Proposed reaction scheme for genipin incubated with primary amines at 37 °C in PBS at pH 7.4. Pathway 1.

The reaction mechanism proposed in Scheme 1 sets the basis for the description of the binding of genipin by small molecules bearing primary amino groups. Numerous contributions, experimental and theoretical [17,18,19], exist on this topic based on the use of UV-VIS spectroscopy. As commented in the Results section, these spectroscopic data could be of difficult interpretation. We resolve to use circular dichroism for comparing the time-evolution of the spectrum when a chiral amine, i.e., glucosamine, or an achiral one, i.e., ethylamine, are incubated with genipin (Figure 2 and Figure 3). While a positive CD band at 280 nm appears for both molecules, only glucosamine gives rise to a positive CD band at 380 nm (Figure 3). Moving from Scheme 1, we propose two possible pathways for the reaction between genipin and primary amines to account for the results obtained from CD. In the first one (Scheme 2), a reaction takes place between genipin and the primary amine leading to compound **G-N** without oxygen being required. 

The computed UV spectrum for **G-N** shows an absorption band at around 380 nm (Figure S8), in good agreement with the study of Di Tommaso and co-workers [19]. Since we assume that, in genipin, the amount of stereoisomer (S,R,R) is equal to the sum of (S,R,S) and (R,R,S), (R)-**G-N** and (S)-**G-N** in Scheme 2 form a racemic mixture when ethylamine is considered, thus explaining the lack of the CD band at 380 nm. At variance, when glucosamine is used as primary amine model, (R)-**G-N** and (S)-**G-N** form two diastereoisomers, thus accounting for the CD band at around 380 nm. Aside from the possible cross-reactions of amino-modified genipin suggested in the literature [18], we here propose two possible associations among two **G-N** molecules in the presence of oxygen. The first one requires the oxygen mediated oxidation of two compounds **G-N** leading to **2(G-N)_ox_**. In addition, **G-N** could undergo an oxygen mediated oxidation and a dehydration to form the adduct **2(G-N)_dox_**. Both compounds, **2(G-N)_ox_** and **2(G-N)_dox_**, show a computed spectrum compatible with the UV peak at around 600 nm (Figure S8) and a complete lack of chiral centers, and thus of CD bands. 

**Scheme 3:**
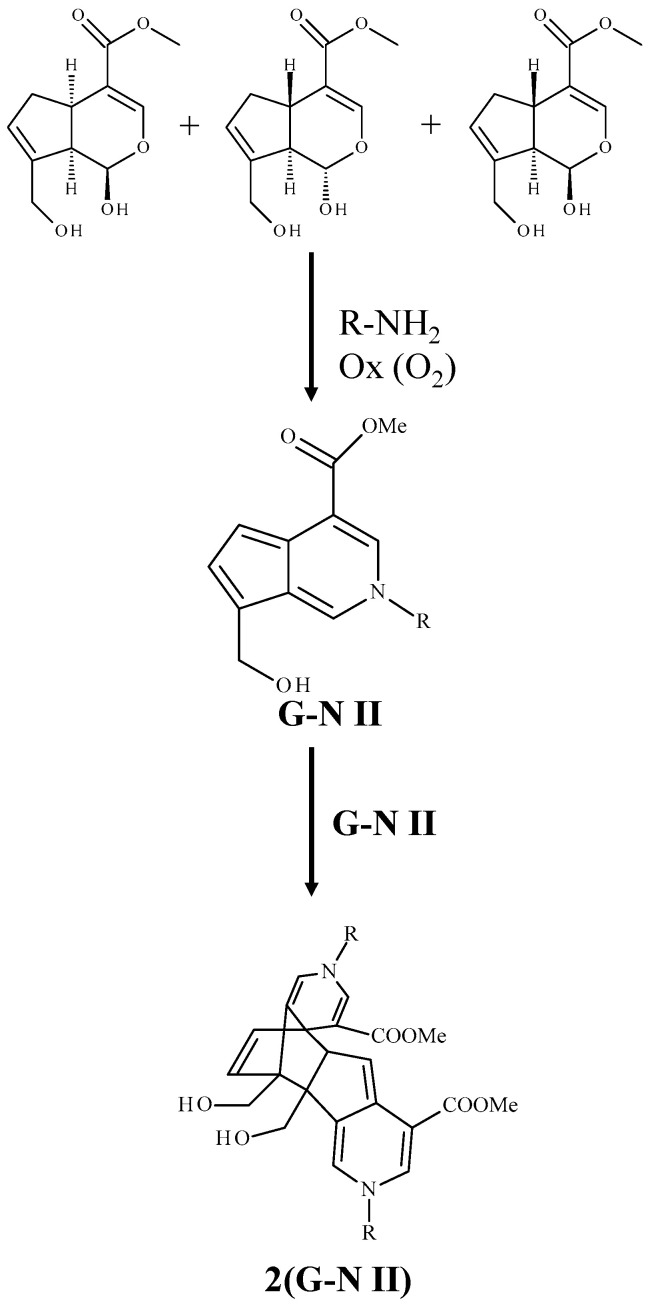
Proposed reaction scheme for genipin incubated with primary amines at 37 °C in PBS at pH 7.4. Pathway 2.

The second pathway (Scheme 3) comprises the reaction of genipin with primary amines in an oxidizing media for the presence of O_2_ and leading to compound **G-N II**. The latter, completely devoid of chiral centers, shows a computed UV-VIS spectrum with peaks at around 290 and 430 nm (Figure S9). 

Since **G-N II** is not expected to give rise to a (positive) CD peak, an additional reaction is foreseen, leading to a molecule with chiral carbon atoms but devoid of a large delocalization of electrons as no blue color closely follows the formation of the CD band at 280 nm. The presence of the cyclopentadiene moiety on **G-N II** could account for a Diels–Alder reaction [33,34,35], with another molecule of **G-N II** leading to the dimer. Tentatively, the Diels–Alder dimer is proposed to be **2(G-N II)**, which shows a computed UV-VIS peak and a CD band at around 280 nm (Figure S9). Although additional analyses will be performed along this line, Diels–Alder reactions involving genipin modified with ammonia have been previously reported in the literature [36]. 

When incubation occurs in oxygen-depleted conditions, both pathways leading to **G-II** and **2(G-N)** are impaired. As a consequence, the CD band at 280 nm and the UV band at 600 nm do not manifest. It follows that only compound **G-N** forms and it is detected in the CD spectrum at 380 nm when glucosamine is used as the primary amine (Figure 2).

**Figure 7:**
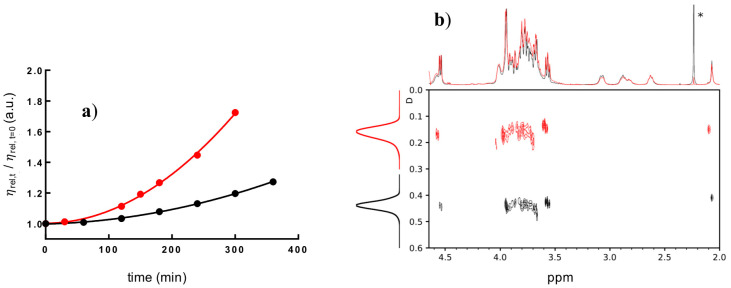
(**a**) Time dependence of the ratio between the relative viscosity at time *t* and the initial relative viscosity for CTL (3.75 g/L) incubated with genipin at concentration of 0.065 mM (black) and 0.13 mM (red) at 37 °C in PBS at pH 7.4. Lines are drawn to guide the eye. (**b**) Overlapped 2D DOSY maps for _r_CTL before (black contours) and after reaction with genipin (red contours) along with the relevant 1D ^1^H-NMR spectra on top and diffusion projections on the left side. The diffusion coefficients (D) are reported in 10^−10^ m^2^s^−1^ units. The asterisk indicates a species fast moving and not linked to the polymer (acetone).

In view of the reaction schemes proposed so far, we now consider the description of genipin binding by CTL in physiological conditions. When oxygen is present, both reactions depicted in Scheme 2 and Scheme 3 take place, hence both peaks at 280 and 380 nm emerge over time (Figure 4). Focusing on Scheme 3, the formation of the compound **2(G-N II)** requires that two genipin molecules bind two distinct CTL chains through a Diels–Alder reaction. This hypothesis accounts for the second-order kinetics of the peak at 280 nm both on cross-linking agent and polymer concentration (Equation (3)) (Figure 5a,c). This conclusion is further strengthened by Figure 7a, which shows a marked increase in the relative viscosity, η_rel_, of the CTL-genipin solution at time points at which the peak at 280 nm is present but no blue color develops. 

The CD peak at around 380 nm (Figure 4c) shows a kinetic of the first order for both genipin and CTL concentrations, pointing to the fact that this CD signal is not related to an immediate reticulation among the polymer chains, but rather to the formation of **G-N** in Scheme 2. In the presence of oxygen, part of **G-N** turns into **2(G-N)_ox_** and **2(G-N)_dox_** leading to a blue color and additional cross-links in the CTL network. However, topological restrictions prevent, to some extent, cross reactions among two **G-N** residues on the polymer and, as a consequence, the UV signal at around 600 nm is less intense than the one recorded for glucosamine at the same amount of primary amines in solution (Figure S6). When the incubation of genipin with CTL is performed in oxygen-depleted conditions for 24 h, the blue color developed is even lower (Figure S4c) as the cross reactions among **G-N** compounds are impaired by the lack of the oxidant. When the sample is exposed to oxygen again, topological restriction by the non-negligible amount of **2(G-N II)** reticulations further limits the amount of cross reaction among **G-N** groups. As a consequence, the UV-VIS signal at 600 nm of the CTL-genipin solution shows no increase over time when exposed to oxygen after 24 h of incubation under oxygen-depleted conditions.

Genipin binding by CTL was also studied by NMR using a polymer sample with reduced molecular weight (_r_CTL). The ^1^H-NMR spectrum of both starting _r_CTL and genipin-_r_CTL is dominated by the resonances of the side chains, which originate narrow signals at variance with the chitosan skeleton, that gives rise to much broader resonances, hard to detect [3]. As an overall, the ^1^H-NMR spectrum after reaction looks almost unchanged and we could not observe any new signal clearly attributable to genipin links, probably because of the synergetic adverse effects of the low molar ratio between genipin/CTL monomeric unit and of the large line width of the NMR signals of nuclei of atoms embedded in polymeric chains. Only the envelope of resonances in the region 4.0–3.5 ppm has slightly changed (Figure 7b and Figure S10). This small variation may be due to the appearance of new signals but also, and more probably, to a conformational change of the polymer upon further chain reticulation. Therefore, the evidence of the successful formation of links by the reaction with genipin is gained through the 2D DOSY maps. The DOSY experiment is employed to measure the self-diffusion coefficient (D) of solutes. It appears clear that the diffusion coefficient of the reaction product is much lower than that of the unreacted _r_CTL (Figure 7b). Since D is inversely related with molecular size, this indicates a remarkable reticulation of the polymer owing to genipin cross-links. Inverse power law between D and molecular weight are reported in literature for polymers [37,38]. Considering an exponent of 0.5, which is reasonable for polysaccharides [38], an inverse relation between the molecular weight ratio and the square of the D ratio can be envisaged. The D of starting _r_CTL peaks around 0.44 10^−10^·m^2^s^−1^ and that after reaction around 0.16 10^−10^ m^2^s^−1^ point to a seven- to eight-fold increase of molecular weight.

## 4. Materials and Methods

### 4.1. Materials

Chitosan was purchased from ChitiNor AS (Sollidalen, Norway). The composition was determined by means of ^1^H-NMR and resulted: GlcNH_2_ = 86% and GlcNAc 14% (MW_RU_ = 167 g/mol). The intrinsic viscosity ([η] = 920 mL/g) was determined by means of capillary viscometry [39], whereas the weight average molecular weight (${\bar{M}}_{w}=306,000\;\pm \;15,000;\;\mathrm{PI}\;=\;1.61\;\pm \;0.08$) was determined by means of SEC-MALS analyses [40]. Picoline-borane complex, deuterated water, sodium nitrite, sodium hydroxide, phosphate buffered saline (PBS), lactose monohydrate, and glucosamine hydrochloride were purchased from Sigma-Aldrich (Chemical Co. USA). Sodium chloride and hydrochloric acid were purchased from Carlo Erba (Cornaredo, Italy). Ethanol/Isopropanol (ETA/IPA) mixture 70/30 *v*/*v* was purchased from Chimen S.R.L. (San Donà di Piave, Italy). Genipin (purity 98%) was purchased from Challenge Bioproducts Co., Ltd. (Yun-Lin Hsien, Taiwan). Deionized water was used in all preparations.

### 4.2. Synthesis of Lactose Modified Chitosan (CTL)

Hydrochloride form of lactose-modified chitosan (CTL) was synthesized as follows in a 5 L reactor. First, 55 g of lactose monohydrate were dissolved in 400 mL of deionized water at *T* = 40 °C under mechanic stirring. After complete dissolution of lactose, 12.5 g of chitosan and an aqueous solution of glacial acetic acid (3.7 M) were loaded into the reactor and the temperature was increased to 60 °C. The acidic solution decreases the pH of the reaction mass, allowing for the complete solubilization of chitosan. After 30 min, a solution of ETA/IPA (70/30 *v*/*v*, 50 mL) containing picoline borane complex (100 mmol) previously dissolved was loaded dropwise in the reactor and the mixture was mechanically stirred for 4 h at *T* = 60 °C. The solution was cooled to room temperature, and 150 mL of aqueous HCl solution (0.86 M) were added. After 30 min, the product was precipitated by dropwise addition of ETA/IPA (70/30 *v*/*v*, 750 mL). The resulting precipitate was extensively rinsed with ETA/IPA (70/30 *v*/*v*) to remove water, air dried and vacuum dried over P_2_O_5_. The final content of water was found to be lower than 8%. The composition of the lactose-modified chitosan (CTL) hydrochloride form was determined by ^1^H-NMR and resulted: GlcNAc = 14%; GlcNH_2_⋅HCl = 24%; GlcLac⋅HCl (*N*-alkylated glucosamine) = 62% (Figure S2). The intrinsic viscosity ([η]) of the lactose-modified chitosan was 480 mL/g.

### 4.3. Synthesis of Lactose-Modified Chitosan at Reduced Molecular Weight (_r_CTL)

Chitosan was degraded following the procedure previously reported [41,42]. The molecular weight of the degraded chitosan was calculated by means of intrinsic viscosity measurements ([η] = 133 mL/g), and resulted 40,000 [39]. The degraded chitosan was used for the preparation of the lactose-modified chitosan at reduced molecular weight (_r_CTL) using the procedure reported in Section 4.2.

### 4.4. Binding of Genipin by Lactose-Modified Chitosan (CTL)

Lactose-modified chitosan solutions in PBS at pH 7.4, final polymer concentration in the range 1.9–7.5 g/L were added of genipin in PBS at different final concentrations in the range 0.04–0.37 mM. The resulting solution was incubated at 37 °C in the presence of oxygen. Typically, 30 mL of solution containing CTL and genipin were incubated in a cylindrical vessel of 60 cm^3^. At selected time points, an aliquot of the CTL-genipin mixture was collected and analyzed by means of different techniques. In the case of measurements in oxygen-depleted conditions extending over 4 h, prior to the incubation at 37 °C, the solution of CTL and genipin was ice cooled to slow down the reaction [43], and extensively degassed using a nitrogen flow. For oxygen depleted conditions over 24 h, the solution of CTL and genipin was incubated in a Thermo Scientific Oxoid 2.5 L jar containing an Oxoid AnaeroGen 2.5 L sachet. Glucosamine and ethylamine were used for comparison.

### 4.5. Intrinsic Viscosity Measurements

Intrinsic viscosity of chitosan and CTL was measured at *T* = 25 °C by means of a Schott-Geräte AVS/G automatic measuring apparatus and an Ubbelohde-type capillary viscometer (Ø = 0.53 mm). A buffer solution composed of 20 mM AcOH/AcNa, pH 4.5, and 100 mM NaCl was used as solvent [41]. The intrinsic viscosity [*η*] values were determined by analyzing the polymer concentration dependence of the reduced specific viscosity (*η_sp_*/*c*) and of the reduced logarithm of the relative viscosity (*ln*(*η_rel_*)/*c*) using the Huggins (Equation (5)) and Kraemer (Equation (6)) equations, respectively:

(5) $$\frac{\eta_{sp}}{c}=\left[\eta \right]+k^{\prime }{\left[\eta \right]}^{2}c,$$

(6) $$\frac{ln\left(\eta_{rel}\right)}{c}=\left[\eta \right]-k^{\prime \prime }{\left[\eta \right]}^{2}c$$

where *k′* and *k″* are the Huggins and Kraemer constants, respectively.

The binding of genipin to CTL in PBS at pH 7.4 was followed at *T* = 37 °C by measuring the relative viscosity of the solutions at different time points with a capillary viscometer.

### 4.6. ^1^H-NMR

Chitosan and CTL were analyzed at *T* = 80 °C by means of ^1^H-NMR spectroscopy carried out on a 400-MR Varian NMR spectrometer operating at 400 MHz. Samples were prepared according to the procedure reported elsewhere [41]. In the case of _r_CTL, the ^1^H-NMR diffusion measurements were carried out at 37 °C on a Varian 500 VNMRS spectrometer (11.74 T) operating at 500 MHz for ^1^H, equipped with a model L650 Highland Technology pulsed field gradient amplifier (10 A) and a standard 5 mm indirect detection, pulsed field gradient (PFG) probe. The final concentrations of _r_CTL and genipin resulted 3.75 g/L and 0.26 mM, respectively. An enhanced stimulated echo pulse sequence with spin lock [44] was employed, with 15 different z-gradient strengths, Gz, between 2 and 54 G/cm, a pulsed gradient duration, δ, of 2 ms, and a diffusion interval, Δ, of 500 ms. The gradients were calibrated on the value of D = 1.90 × 10^−9^ m^2^ s^−1^ for ^1^H in D_2_O at 25 °C (99.9%) [44]. Then, 90% D_2_O with PBS buffer was used as solvent and proton chemical shifts are referred to internal standard trimethylsilylpropanesulfonate (DSS). Solvent suppression was accomplished by presaturation. The data were processed as diffusion ordered spectroscopy (DOSY) [45] spectra by means of the relevant routine of the OpenVnmrJ software, version 2.1 (https://openvnmrj.org/)

### 4.7. Circular Dichroism (CD)

Circular dichroism spectra of the CTL-genipin solutions in PBS buffer at pH 7.4 were recorded at different time points using a Jasco J-700 spectropolarimeter in the wavelength range 220–450 nm or 220–700 nm at *T* = 25 °C. A quartz cell of 1 cm optical path length was used, and the following setup was maintained: bandwidth 1 nm; time constant 2 s; scan rate 100 nm/min. Two spectra, corrected for the background, were averaged for each sample. 

### 4.8. UV-VIS Measurements

UV-VIS spectra of the CTL-genipin solutions in PBS at pH 7.4 were recorded at different time points at *T* = 25 °C in the range from 220 to 700 nm with a Shimadzu UV-2450 spectrophotometer. UV absorption of CTL-genipin solutions in PBS at pH 7.4 incubated, in the presence or in the absence of oxygen, at 37 °C for 24 h, was recorded with a multiplate reader FLUOStar Omega-BMG Labtech. Then, 150 µL of solution were transferred into a 96-wells multi-well and the absorbance was measured at 600 nm. Eight replicates, corrected for the background, were averaged for each data point.

### 4.9. Computational Details

Molecules were modeled and their structure energy minimized using the Avogadro program [46] and applying the MMFF94 force field method. Theoretical calculations were carried out using time-dependent DFT of the ORCA package (4.0). Computations on molecules were carried out using the B3LYP hybrid functionals with def2-TZVP basis set with RIJCOSX acceleration as a compromise between accuracy and reasonable calculation speed [47]. The solvent effect was computed by the CPCM-SDM model using the water static dielectric constant ε = 80.4 and the refractive index *n* = 1.33.

## 5. Conclusions

Genipin is largely used, in particular, in combination with chitosan, for hydrogel and scaffold preparation in tissue engineering and drug delivery fields. Yet, the mechanism of binding has only been partially elucidated and it has been overlooked when physiological conditions (pH 7.4) are considered. To this end, a lactose-modified chitosan soluble at all pH values has been considered in the present work. An extensive use of the circular dichroism spectroscopy allowed underlining novel features for both the auto-reaction of genipin and its binding to primary amines. In particular, the role of oxygen as an oxidizing agent is elucidated and the effect of its depletion explained. The mechanism for the binding of primary amines by genipin assigns a central role to the compound **G-N** that, in the presence of oxygen, undergoes two oxidation pathways. Both the latter, when lactose-modified chitosan is considered, lead to network formation. While the first oxidation process accounts for the well-known blue coloring of the solution, the second oxidation is here proposed, leading to a Diels–Alder reaction (**2(G-N II)**). The latter accounts for the marked increase of the relative viscosity of lactose-modified chitosan prior to the blue coloring of the solution. The analyses of the reaction kinetics between lactose-modified chitosan and genipin confirm the proposed mechanism. Finally, the comparison of the CD spectra between ethylamine and glucosamine allows proposing that the compound **G-N** is a racemic mixture for the former primary amine and that the genipin used in this work is mainly formed by three stereoisomers. The products obtained from genipin binding to CTL in the presence of oxygen display hydrophobic domains; it will be important to evaluate their effect on the physical-chemical properties of the hydrogel and its interaction with cells.

## Supplementary Materials

Supplementary materials can be found at https://www.mdpi.com/1422-0067/21/18/6831/s1. Figure S1. Proposed reaction schemes for chitosan treated with genipin as reported by Butler et al.; J. Polym. Sci.: Part A: Polym: Chem.; 2003; 41; 3941–3953; Figure S2. Structure of lactose modified chitosan (CTL); Figure S3. Calibration curve for genipin in PBS at pH 7.4 reported as ellipticity of the CD signal at 240 nm vs. genipin concentration; Figure S4. (a) Time-dependence of the ellipticity at 380 nm for CTL at a concentration 1.9 g/L (black), 3.75 g/L (red) and 7.5 g/L (blue) in the presence of genipin at a concentration of 0.37 mM. (b) Dependence of the UV-VIS signal at 600 nm from CTL concentration in the presence of genipin at 0.37 mM after 24 h of incubation; Figure S5. Time dependence of the ellipticity at 380 nm for CTL treated with genipin (0.37 mM) in the presence of oxygen (black) or in oxygen-depleted conditions (red); Figure S6. UV-VIS spectrum of genipin treated with glucosamine (black) and CTL (red) for 6 h; Figure S7. Computed CD (a) and UV-VIS (b) spectra for (S,R)-GII and (R,R)-GII; Figure S8. Computed UV-VIS spectrum of G-N (a), 2(G-N)ox (b) and 2(G-N)dox (c); Figure S9. Computed UV-VIS spectrum of G-N II (a) and 2(G-N II); Figure S10. (a–c) ^1^H-NMR spectra at different scale of rCTL before (black) and after (red) reaction with genipin.
