# Supplementary material for: On the Mechanism of Genipin Binding to Primary Amines in Lactose-Modified Chitosan at Neutral pH
Source: Int J Mol Sci. 2020 Sep 17;21(18):6831. doi: 10.3390/ijms21186831 (PMC7554727; doi:10.3390/ijms21186831)
Supplement: Supplementary file 1 [file ijms-21-06831-s001.pdf]

Supplementary Materials

On the mechanism of genipin binding to primary amines in lactose-modified chitosan at neutral pH

Chiara Pizzolitto [a], Michela Cok [b], Fioretta Asaro [c], Francesca Scognamiglio [a], Eleonora Marsich [a], Francesco Lopez [d], Ivan Donati [b], and Pasquale Sacco [b,*]

[a] Department of Medical, Surgical, and Health Sciences, University of Trieste, piazza dell'Ospitale 1, 34127 Trieste, Italy.

[b] Department of Life Sciences, University of Trieste, via Licio Giorgieri 5, 34127 Trieste, Italy.

[c] Department of Chemical and Pharmaceutical Sciences, University of Trieste, via Licio Giorgieri 1, 34127 Trieste, Italy.

[d] Department of Agricultural, Environmental and Food Sciences (DiAAA), University of Molise, Via De Sanctis, I-86100 Campobasso, Italy

*** Corresponding Author**
Tel: +39 040 558 8733
e-mail: psacco@units.it

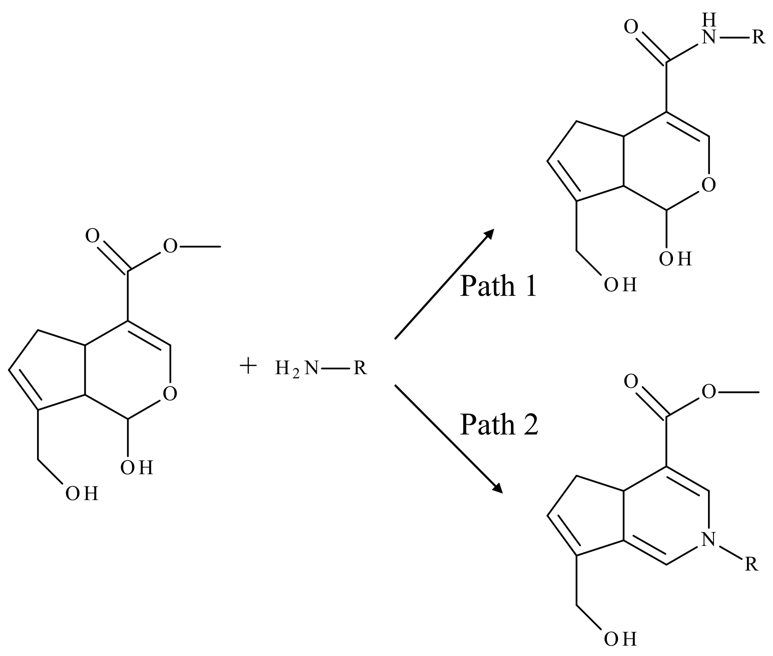

Figure S1. Proposed reaction schemes for chitosan treated with genipin as reported by Butler *et al.*; *J. Polym. Sci.: Part A: Polym: Chem.*; **2003**; *41*; 3941-3953.

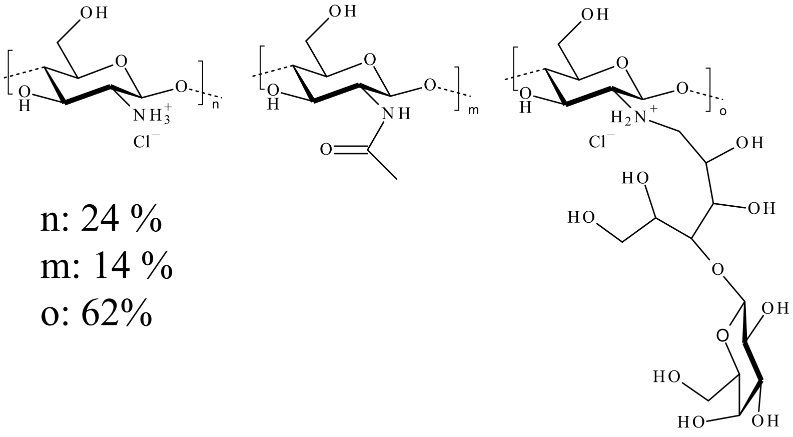

Figure S2. Structure of lactose modified chitosan (CTL).

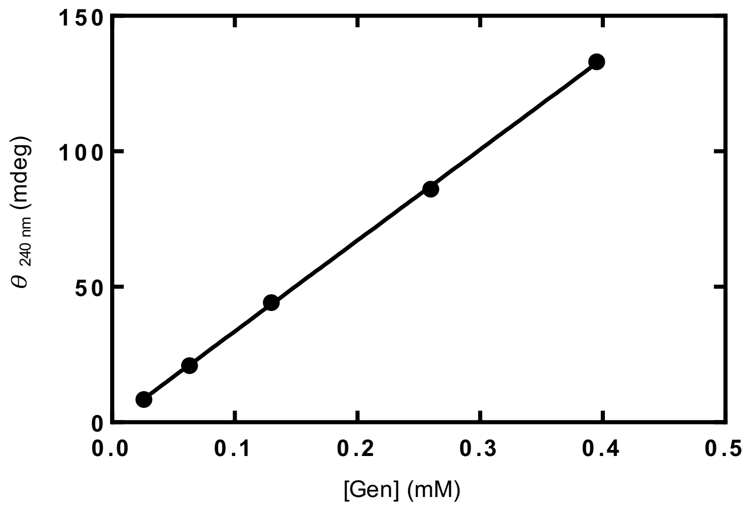

Figure S3. Calibration curve for genipin in PBS at pH 7.4 reported as ellipticity of the CD signal at 240 nm *vs*. genipin concentration.

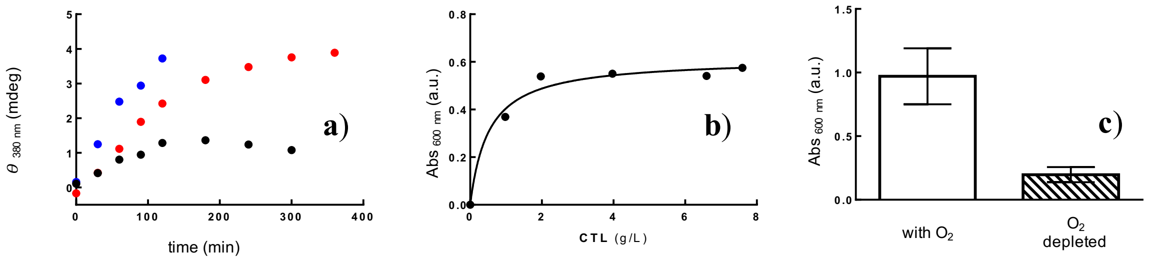

Figure S4. **a**) Time-dependence of the ellipticity at 380 nm for CTL at a concentration 1.9 g/L (black), 3.75 g/L (red) and 7.5 g/L (blue) in the presence of genipin at a concentration of 0.37 mM. **b**) Dependence of the UV-VIS signal at 600 nm from CTL concentration in the presence of genipin at 0.37 mM after 24 h of incubation. In **a**) and **b**) the incubation was performed in the presence of oxygen. **c**) Absorbance measured at 600 nm for CTL (3.75 g/L) incubated with genipin (0.13 mM) for 24 h in the presence of oxygen and in oxygen-depleted conditions for 24 h. In all cases, the incubation was performed at 37 °C in PBS at pH 7.4.

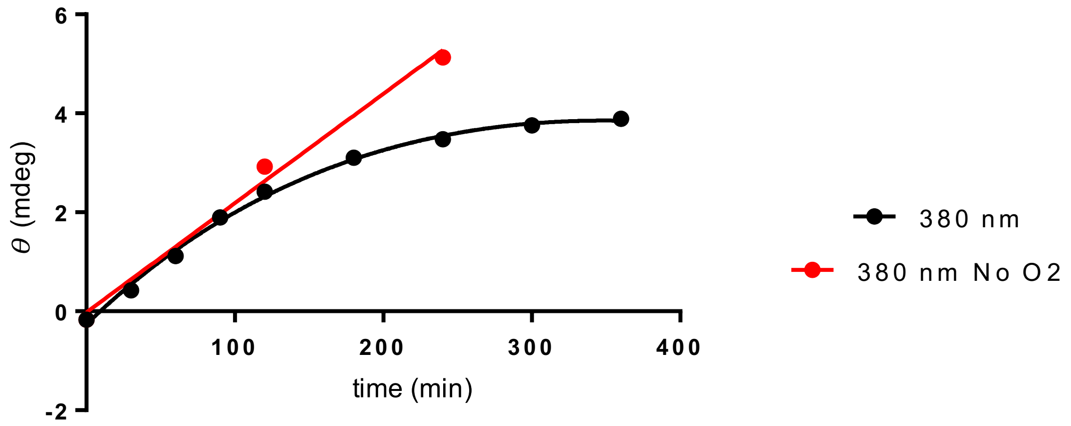

Figure S5. Time dependence of the ellipticity at 380 nm for CTL treated with genipin (0.37 mM) in the presence of oxygen (black) or in oxygen-depleted conditions (red). The incubation was performed at 37 °C in PBS at pH 7.4. CTL concentration was 3.75 g/L.

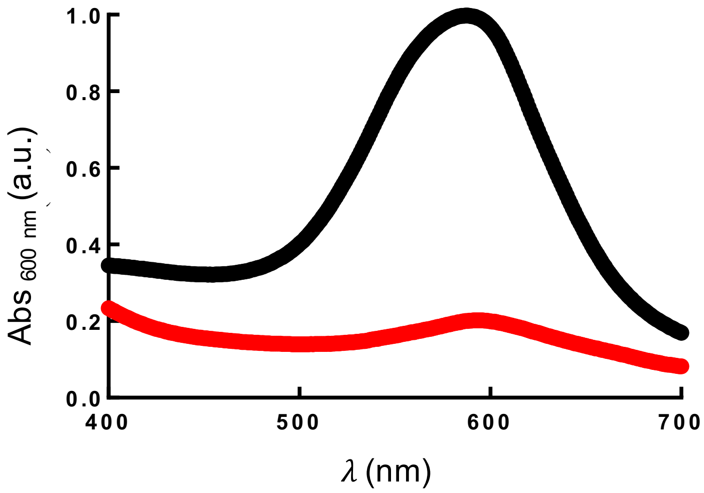

Figure S6. UV-VIS spectrum of genipin treated with glucosamine (black) and CTL (red) for 6 h. In both cases, the concentration of primary amino groups was 1 mM. The incubation was performed in the presence of oxygen at 37 °C and in PBS at pH 7.4.

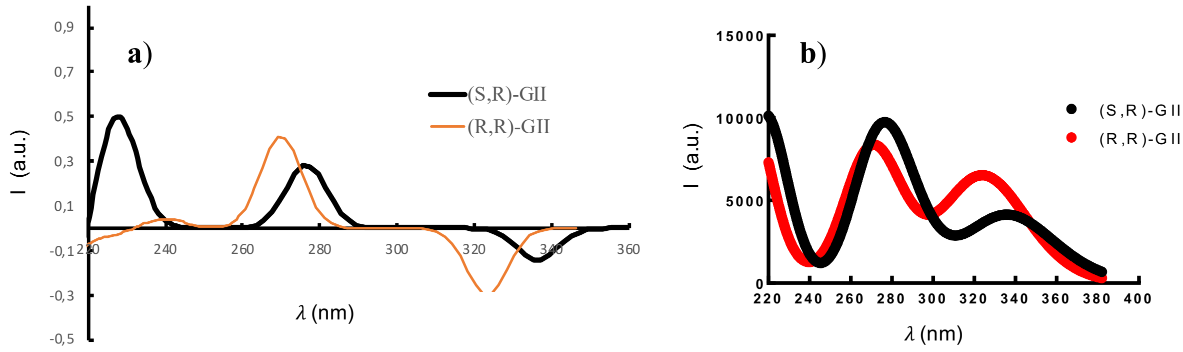

Figure S7. Computed CD **a**) and UV-VIS **b**) spectra for (S,R)-**GII** and (R,R)-**GII**. The geometrical optimization of the molecule was performed using Avogadro software applying MMFF94 force field method. UV-VIS and CD spectra were calculated with ORCA package.

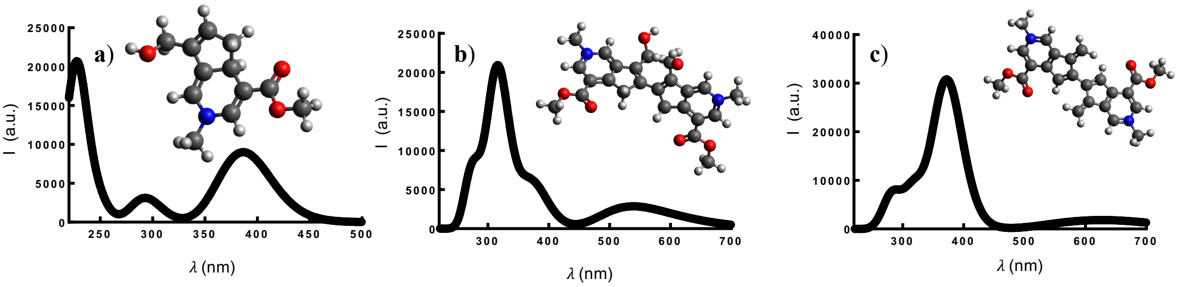

Figure S8. Computed UV-VIS spectrum of **G-N** (**a**), **2(G-N)ox** (**b**) and **2(G-N)dox** (**c**). The geometrical optimization of the molecule was performed using Avogadro software applying MMFF94 force field method. UV-VIS spectra were calculated with ORCA package.

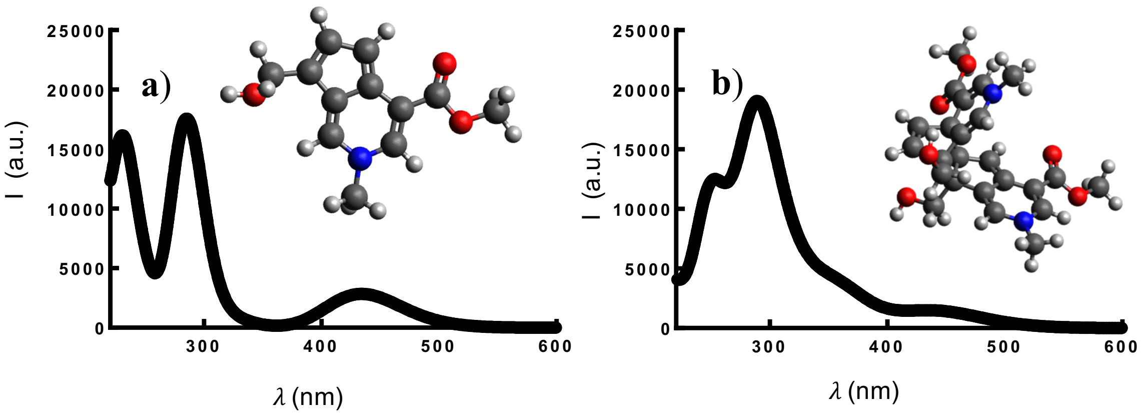

Figure S9. Computed UV-VIS spectrum of **G-N II** (**a**) and **2**(**G-N II)**. The geometrical optimization of the molecule was performed using Avogadro software applying MMFF94 force field method. UV-VIS spectra were calculated by means of ORCA package.

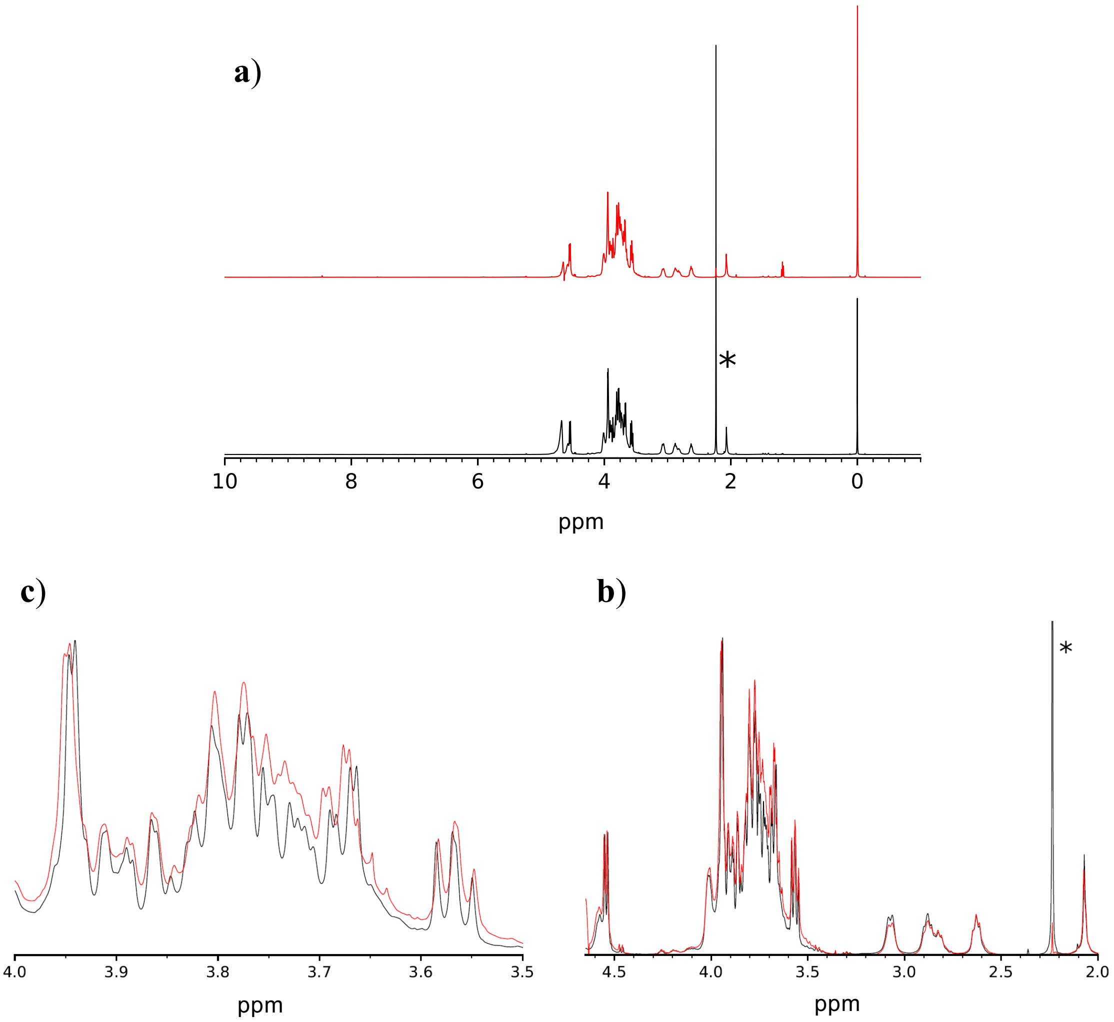

Figure S10. **a,b,c**) ^{1}H-NMR spectra at different scale of $_{r}$CTL before (black) and after (red) reaction with genipin. The asterisk indicates a species fast moving and not linked to the polymer (acetone).
